# History of cardiopulmonary resuscitation in ancient China: a narrative review

**DOI:** 10.1186/s13019-020-1086-5

**Published:** 2020-03-23

**Authors:** Yang Yu, Xiaojie Liu, Li Juan Wang, Sudena Wang, Hushan Ao

**Affiliations:** National Center For Cardiovascular Diseases, Fuwai Hospital Chinese Academy Of Medical Sciences, Chinese Society of Cardiothoracic and Vascular Anesthesiology, Beijing, China

**Keywords:** Chinese medicine, Cardiopulmonary resuscitation, Zhongjing Zhang, Artificial respiration

## Abstract

Modern cardiopulmonary resuscitation (CPR) comprises an open airway, artificial ventilation, chest compressions and, if necessary, defibrillation. CPR has been intensively studied and tested to perfect an integrated and effective resuscitation system in the West. However, CPR efforts in China has been understudied and underreported. CPR has been performed for more than 2000 years in China. As early as the third century BC, a Chinese doctor named Zhongjing Zhang presented a detailed program to save patients from suicide by hanging in the book entitled “Synopsis of the Golden Chamber”. Dr. Zhang proposed “not only to save the body, but also to save the spirit”, which remains a guiding principle in modern resuscitation: to not only ensure cardiopulmonary recovery but also preserve the brain function. We aim to review and summarize efforts of CPR in China from a historic point of view.

## Introduction

We will review developments of cardiopulmonary resuscitation in ancient China by dynasty to provide a historic perspective.

### The first cardiopulmonary resuscitation was reported in the Han dynasty (202 BC ~ 220 AD)

**Fig. 1:**
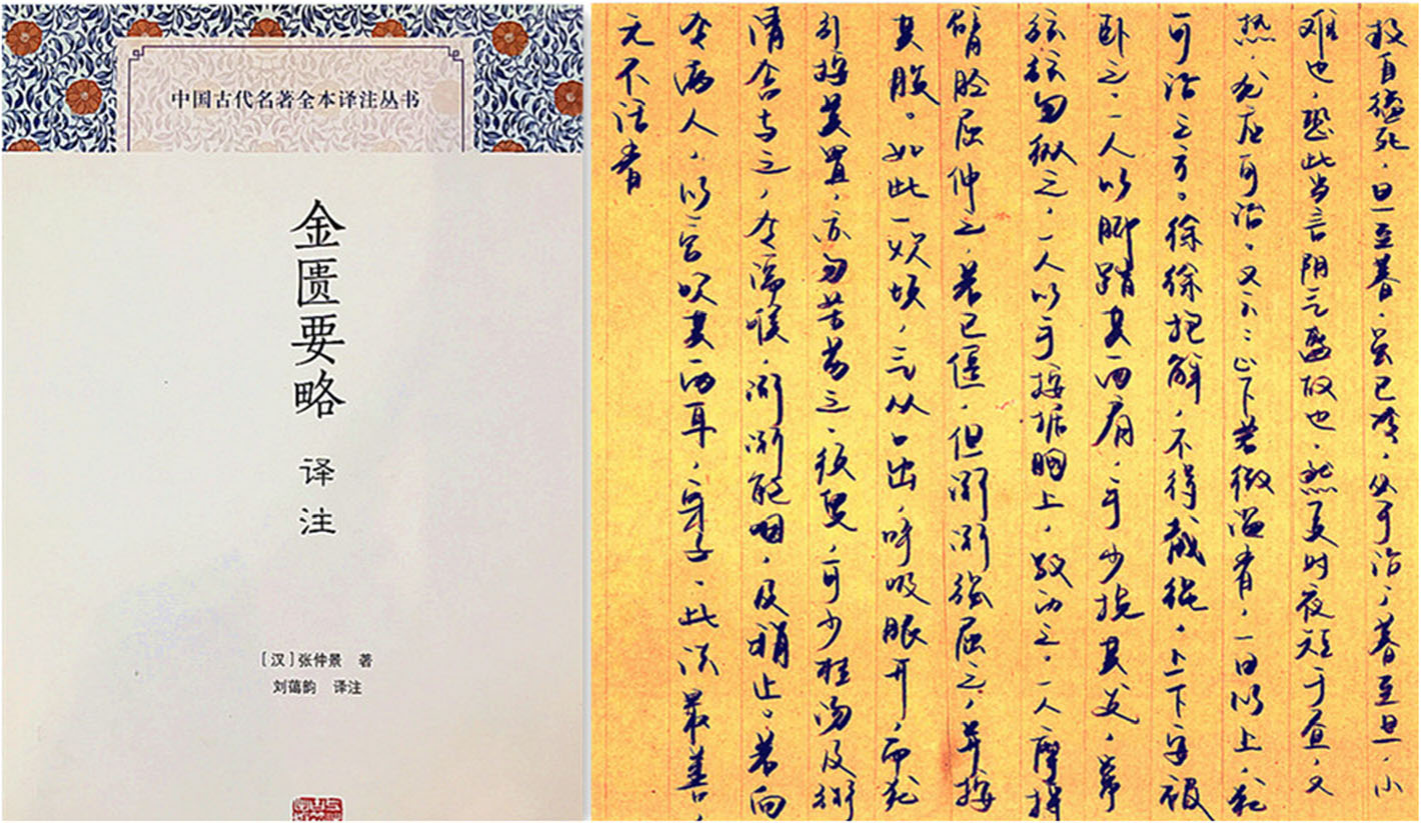
Synopsis of the Golden Chamber

In the Han Dynasty, Zhongjing Zhang (about 145 ~ 208 BC) wrote “Synopsis of the Golden Chamber”. (See Fig. 1, the cover of “Synopsis of the Golden Chamber” and the copy of original text.) He described saving a hanged man from dying by slowly embracing them and then untying the rope without cutting it. Their body should be covered top to bottom with a quilt. One person shall put his feet on the victim’s shoulders and gently raised his head by pulling without dragging the hair. The second person should press continuously on the chest with his hands. The third person shall massage the arms and legs into extended positions. This was to allow the limbs to gradually bend and flex. Alternatively, they might try pressing the victim’s belly if the limbs remained stiff. After the period of about 30 min, the victim should open their eyes and breathe through their mouth. If the victim did not respond, it was time to stop [1]. In 1857, Marshall Hall advanced the chest-pressure method, which was modified in 1861 by Silvester to become the chest-pressure arm-lift method in supine patients. Fourteen variants of this technique continued to be practiced with fervor until the 1960s [2]. These two methods of the chest compression are similar, although they are from different countries, this also can confirm that the ancient people are really clever. We need to learn more about the ancient record.

The descendants of Dr. Zhang, Hua Tuo, wrote similar descriptions in the book titled, “Handbook of Hua Tuo” [3]. This detailed description of CPR was about a thousand years prior to the first report in the West. Several key points of cardiopulmonary resuscitation are presented: 1) the victim should be in a flat position, 2) open the airway (by pulling the hair), 3) press the chest, 4) lift the arms for respiration, 5) abdominal compression to push diaphragm up and aid respiration, 6) continue the above operation for about 30 min. This is very impressive considering the lack of physiological knowledge, modern equipments and delays of experienced rescuers to reach the victim.

### Artificial respiration in the Jin (266~420 AD)

**Fig. 2:**
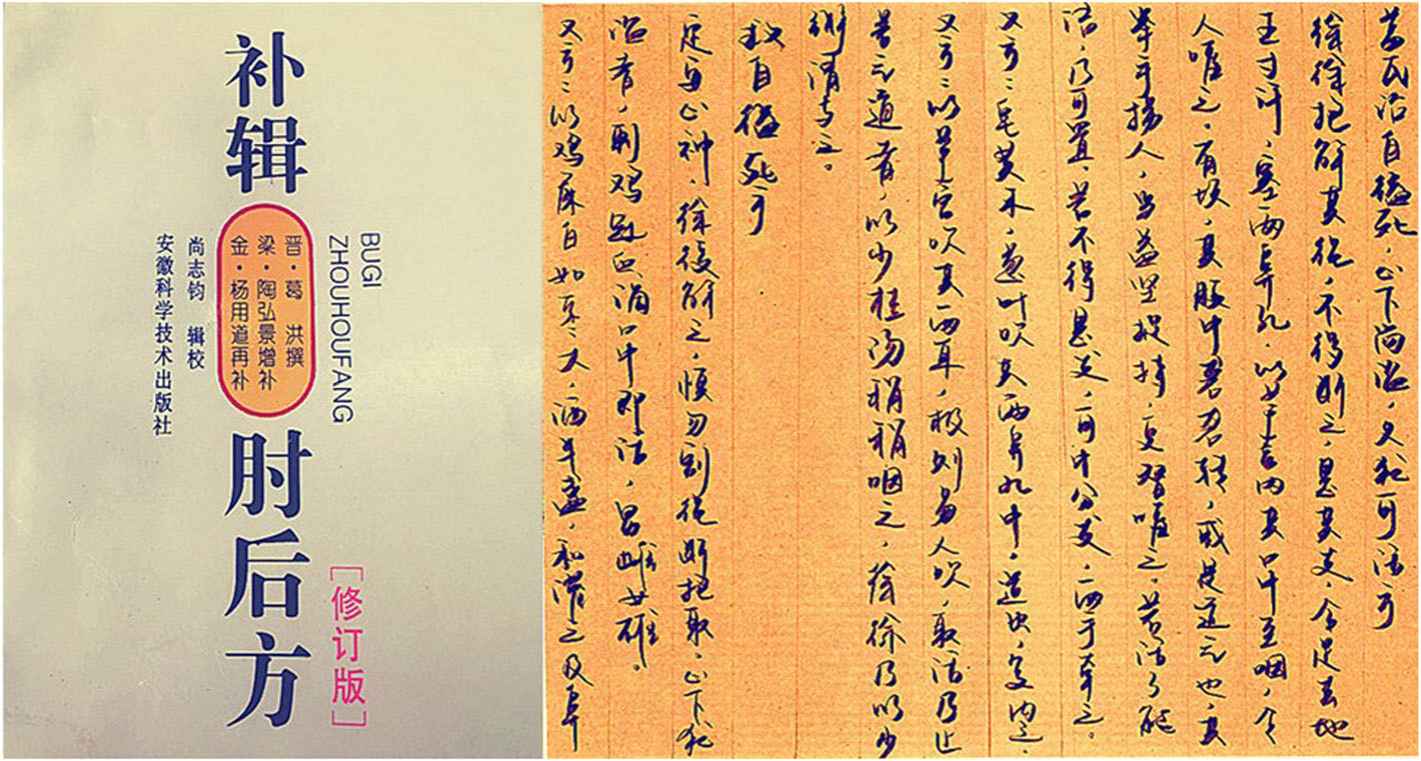
Handbook of Prescriptions for Emergencies

Artificial respiration was emphasized during the Jin Dynasty period. Hong Ge (284 ~ 364 AD) wrote the “Handbook of Prescriptions for Emergencies”.(See Fig. 2, the cover of “Handbook of Prescriptions for Emergencies” and the copy of original text.) In this book, Dr. Ge described that a reed pipe was inserted from the mouth to the throat and another person blew through it after blocking the nostrils. If the patient has borborygmus, the airway may be considered unobstructed. The person who lifted the patient should continue to do so, and not put the patient down. Rescuers shall periodically relieve the one supporting respiration. The process was halted if the patient recovered enough to speak [4]. Although the ancient didn’t explain the theory, we are excited about their wisdom. Dr. Peter Safar, an anesthesiologist, in the mid-twentieth century investigated various techniques for airway management. He found that 50% of patients’ airways would be opened by a head tilt, the remaining 50% could be opened with either thrusting the mandible forward or the insertion of an oropharyngeal airway [5].

Dr. Ge advised rescuers to place the powder of Chinese honeylocust fruits or Chinese crinum leaf into the nostrils. The strong smell caused a sneeze or similar robust exhalation through nasal irritation and helped to recover respiration [3]. This can be repeated several times until the patient can breathe smoothly. When the patient recovered respiration, then drug assisted rescue, such as with the powder of websterite and clove (a kind of traditional Chinese medicine) cooked in a soupwould help them to wake up. This book emphasized resuscitation methods with artificial respiration and this was the basic of modern resuscitation in China.

The resuscitation approaches has been improved in the following aspects during the Jin Dynasty: 1) the patient should be ventilated, 2) the feet were raised more than 17 cm from the ground to return blood to the heart, 3) block the nostrils and ventilate through with a bamboo tube, 4) recovery efforts must be sustained until waking was emphasized, 5) adding a chemical stimulus: placing strong smelling compounds into the nose to make patients sneeze or cough, 6) drug assisted rescue These methods provided positive pressure ventilation. The earliest prototype of the “oropharyngeal airway” was an innovation by Dr. Ge. Today, the more advanced “cuffed oropharyngeal airway” is used in hospitals all over the world. The ancient Chinese physicians combined the steps for first-aid and they paid more attention to artificial respiration in the Jin and Sixth Dynasties.

### Theoretical exploration of CPR in the sui dynasty (581 ~ 618 AD)

**Fig. 3:**
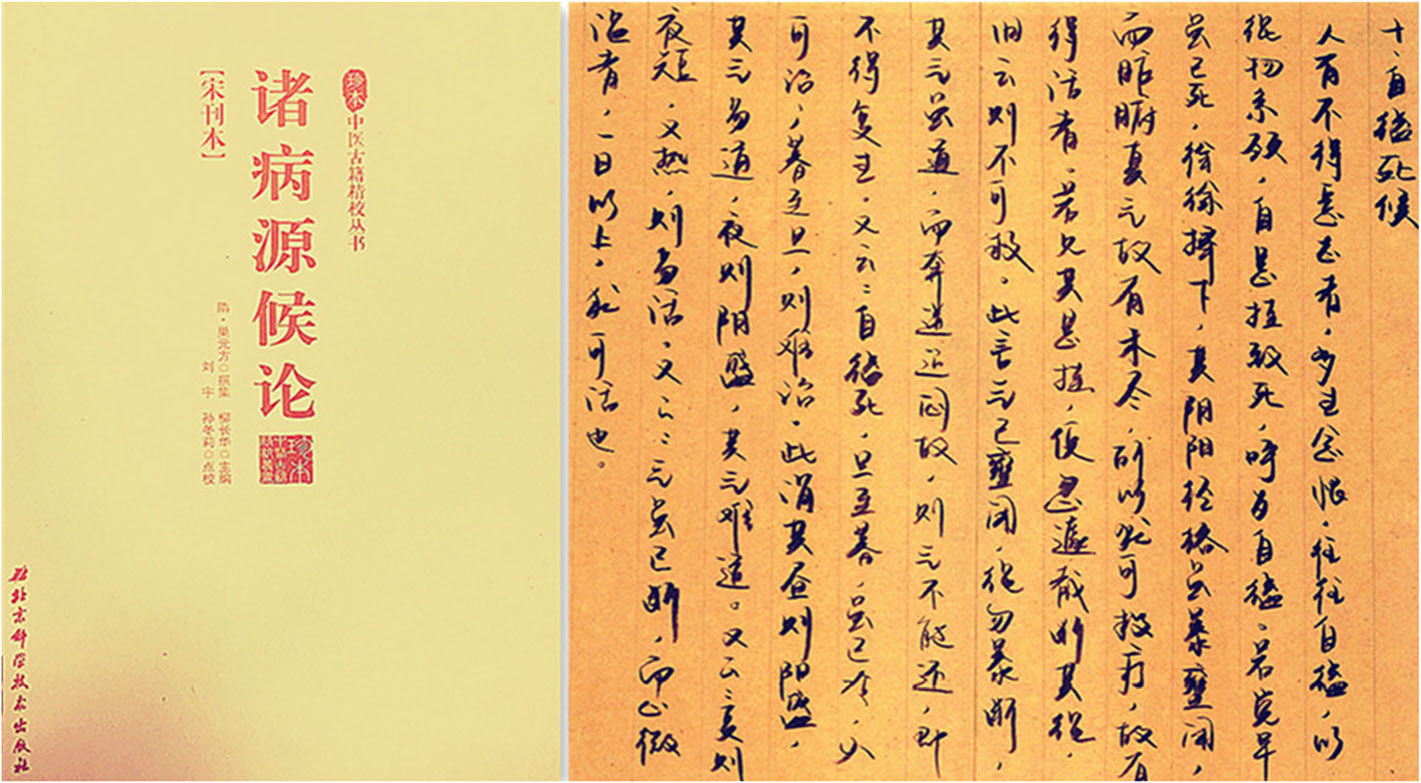
General Treatise on the Cause and Symptoms of Diseases

In the Sui Dynasty, the “General Treatise on the Cause and Symptoms of Diseases” (610 BC) was written by the famous doctor Yuanfang Chao. (See Fig. 3, the cover of “General Treatise on the Cause and Symptoms of Diseases” and the copy of original text.) It is the earliest record of the etiology, pathogenesis and syndromes of diseases in China. It emphasized the theory of resuscitation, again related to saving the hanged man in the chapter “Hanging death syndrome”. Although hanging was known to close the Yin and Yang main and collateral channels, some Yang Qi remained around the viscera, so the hanged man might be saved. Several points were presented: 1) cardiac resuscitation should begin as early as possible, 2) it was equally important to restore breathing and circulation [6].

### CPR in the tang dynasty (618 ~ 907 AD)

**Fig. 4:**
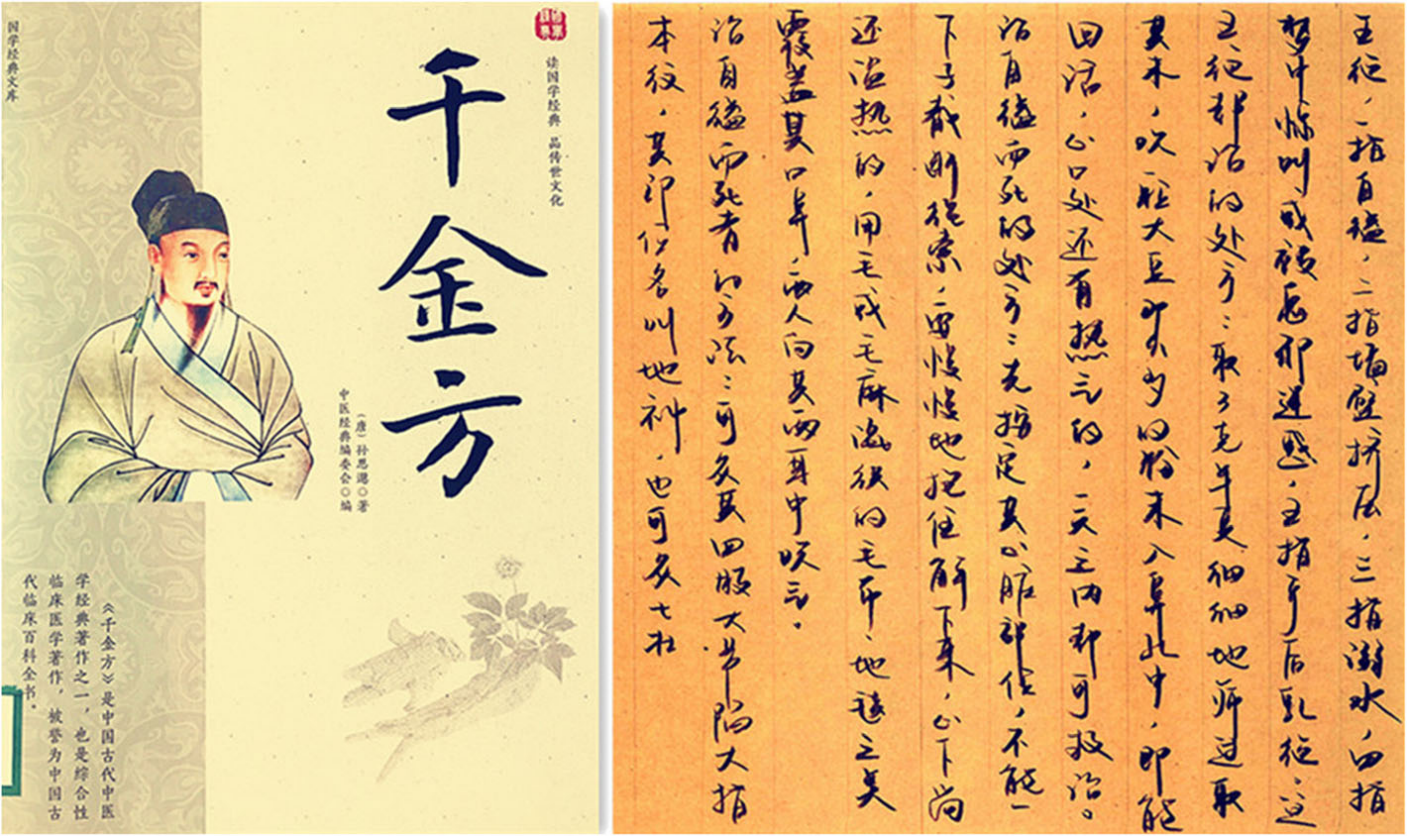
Valuable Prescriptions for Emergencies

Simiao Sun wrote “Valuable Prescriptions for Emergencies” in 651 BC. (See Fig. 4, the cover of “Valuable Prescriptions for Emergencies” and the copy of original text.) To resuscitate a person, it said to lay them flat, plug their ears, and insert a hollow bamboo tube into their mouth. Two people alternately blew into the tube. Efforts were to be ended half a day later if the patient was not resuscitated. Another method had rescuers blow an irritating or strong smelling powder into the nose. As patients recovered consciousness they were fed Lan Qing Juice (a type of traditional Chinese medicine). In a fourth step of resuscitation, rescuers applied acupuncture and moxibustion to points in the limbs [7]. The Tang Dynasty, CPR has been further improved in methods and details: 1) combining acupuncture and moxibustion with CPR, 2) updated the application of pharyngeal intubation to the mouth and replacing the reed tube with bamboo, 3) the resuscitation was extended from 30 min into half a day. Benefitting from previous experiences, Sun’s method of CPR was complemented and improved, although there were some idiosyncrasies, such as “blocking the ears”. In addition, Andreas Vesalius published “De Humani Corporis Fabrica” which describes blowing into a tube to resuscitate an animal around the year 1000. This was also a prototype of an oropharyngeal airway. Still, both of these work had great influences on later generations.

### The development of CPR in the north song dynasty(960~1127 AD)

There is a book named “Zhong Zang Jing”. According to recent research the book should be ascribed to the North Song Dynasty, but the writer is unknown [8]. We used to think the writer was Hua Tuo. But many researchers don’t agree with that, and we don’t have a conclusion. In this book, the writer mentioned that to rescue a hanged person, besides to stimulate with things with strong smell, it is necessary to blow air into the patient’s mouth and the relatives can do this [9]. In the West, in 1472 Paulus Bagellardus published the first known book on childhood diseases and described mouth-to-mouth resuscitation by recommending to midwives to blow in to the new born’s mouth if there is no respiration [10]. All of these history is the basic of mouth-to-mouth resuscitation in modern medical.

### The popularization of CPR in the Ming dynasty (1368~1644 AD)

**Fig. 5:**
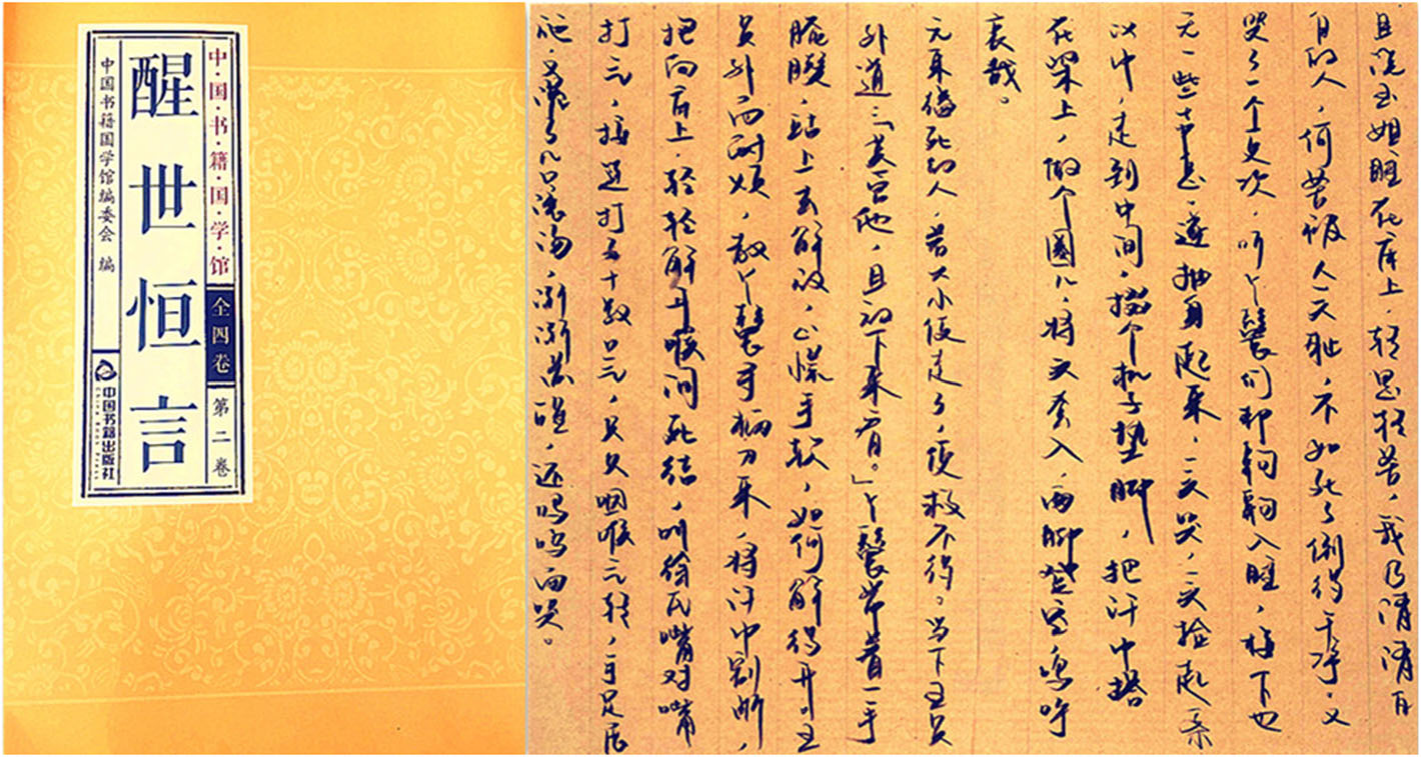
Lasting Words to Awaken the World”

In the Ming Dynasty, Menglong Feng wrote “Lasting Words to Awaken the World” (1627 AD), in which there is a detailed description of mouth-to-mouth resuscitation as it became more popular. (See Fig. 5, the cover of “Lasting Words to Awaken the World” and the copy of original text.) There is a real story in the book “Xiuting Zhang Escaped to Save his Father”. Xiuting Zhang’s fiancée hanged herself because her father disagreed with the engagement. A family member rescued the girl by the method “mouth-to-mouth resuscitation” recorded in this book [11].That implied that about 400 years ago, civilians in China were able to use “mouth-to-mouth resuscitation” to carry out first aid resuscitation. In the West, mouth-to-mouth resuscitation techniques were described by Dominque Jean Larrey, Napoleon’s chief battlefield surgeon. Also, since Napoleon came after 1732, Tossach should come first [12]. The Paris Academy of Sciences officially recommended mouth-to mouth resuscitation from drowning in the year 1740.

### Consistent implementation of CPR in the Qing dynasty(1644 ~ 1912 AD)

In the Qing Dynasty, a number of first-aid books were published, such as “Ancient Valuable Prescription Collection” (1790, AD) by Jin Jiang, “Emergency experience” (1801 AD) by Tingjian Ye, and “First Aid Collection” (1803 AD) by Pengcheng Cheng. The Chinese techniques of cardiopulmonary resuscitation had spread to Japan and were widely accepted. For example, the famous Japanese medical scientist, Danbo Yuen, wrote “Emergency Selection” (1801 AD) and quoted “YuanTi Ji”, which was written by Chinese physicians in the Qing Dynasty.

These treatises emphasized the difficulty of saving a hanged victim, especially if they were hanged with a fine line and cut deeply. In these cases, rescuers should be quick to lift the person and untie the knot without cutting the rope. The rescuers were to rub the ligature mark left by the line, and manipulate the trachea while pressing the chest and belly. Another rescuer performed mouth-to-mouth resuscitation. The third person put his feet on the victim’s shoulders and gently raised his head by his hair. Raising the head should be done quickly and the head should not be lowered. If the arms and legs of the hanged man were rigid, they must be folded, like a monk sitting. Meanwhile, the powder of the *Pinellia ternata* and Chinese Honeylocust fruit were blown into the nostrils with two bamboo tubes. Point stimulation with acupuncture and moxibustion were employed to assist resuscitation. The stomach was infused with strong ginger and Su He Wan made of styrax, benzoin, sandalwood, rhizome cyperi, costustoot, clove, frankincense, *Illicium verum*, cinnabar and bornrol to restore consciousness, borneol and styrax to smooth whole body meridian and others to disperse cold air in the body [13].. Because unconscious patients can’t swallow, the pill was placed into their mouth and the smell led it be swallowed by reflex.

All methods must be continued until the “air is out of the mouth” and their eyes are open. The book “An encyclopedia of Medicine” by Shicheng Gu, added that the person being treated must have heat (Yang Qi) around the heart, no new stools and no protruding tongue. The Qing Dynasty made the following contributions to CPR: 1) the previous experience was incorporated, 2) the resuscitation methods complementary and improved by acupuncture and medicines, 3) the boundaries of resuscitation were clarified, 4) the resuscitation procedures of up to more than 10 steps were promulgated. (a: Hold the patient and untie the rope, don’t cut the rope. b: Knead the trace made by the rope and try to make the weasand to be round. c: Massage chest and abdomen. d: Mouth-to-mouth resuscitation. e: One person shall put his feet on the victim’s shoulders and gently raised his head by pulling without dragging the hair. f: If the patient’s hands and feet have been tetanic, it is necessary to make them hunker, just like monks meditate. g: Placing strong smelling compounds into the nose to make patients sneeze or cough. h: Acupuncture at philtrum. i: Moxibustion Yongquan point. j: Make Su He Wan dissolve into ginger decoction and feed the patient.) 5) the recovery techniques had spread to other countries.

### Popularization of CPR in China

Reviewing the CPR history in ancient China, we have to admit that we have fallen behind in the field of Cardiopulmonary Resuscitation after more than a century. In China, there are more than 230 million people with cardiovascular disease, and 550,000 individuals experience cardiac arrest every year. The survival rate of out-of-hospital cardiac arrest less than 1% in China (compared with 12% in the United States). Early initiation and good quality of cardiopulmonary resuscitation (CPR) by bystanders and automated external defibrillator use are crucial for saving patients in cardiac arrest. However, the implementation rate for bystander CPR in China is low (4.5%in large and medium-sized cities around China, 11.4% in Beijing, and 4,2% in Shanghai,vs 46.1% in the United States, 29% in Canada, 46–73% in Sweden, 32.2% in Japan, and 21.2% in Australia) and the quality is also poor, whichis reflected by the low survival rates following by stander CPR in China [14].At present, CPR is more often implemented in medical schools and hospitals. The public awareness rate outside the hospital is low. Without legal support, the right of rescuers is not protected by the law. Institutions and forms of CPR training are mixed, many irregular training misleads the public. There is no unified and normative training material with the national standard.

**Fig. 6:**
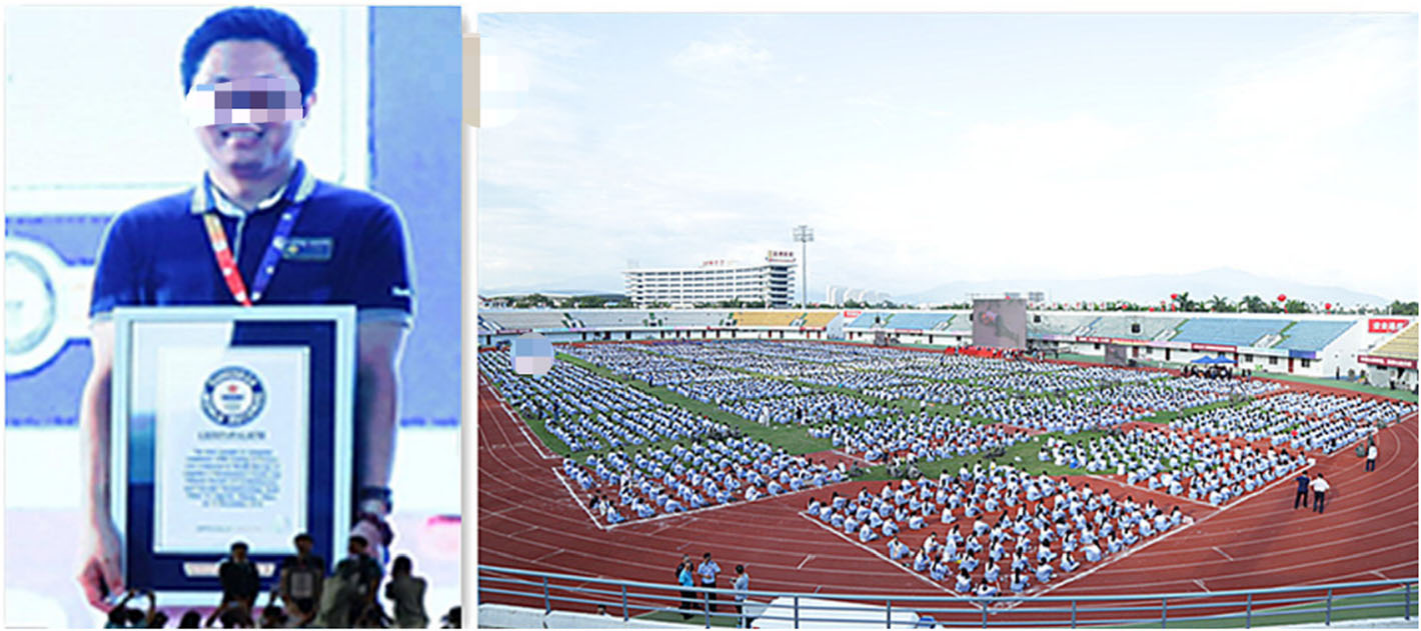
Guinness Book of World Records

However, most of the CPR trainers are part time job, they are under too much pressure of clinical responsibilities and CPR skills need to be regularly evaluated and updated. So that, it is necessary to have an organization with full-time job trainers to do this. CPR training is the most important public welfare activity in the Chinese Society of Cardiothoracic and Vascular Anesthesiology (CSCVA). In July, 2015, they created more activities in order to raise the concern of the whole society on health of the cardiothoracic and vascular systems, and to popularize the scientific standardized knowledge and technology of CPR. This initiative was based on the emergency medicine concept and aimed to popularize the normative method of CPR, increase public participation in this life-saving actions, spread public welfare concepts, maximize lifesaving, reduce mortality, alleviate pain and reduce disability, and improve public health awareness and general knowledge of CPR. Up to now, the Society (CSCVA) has implemented activities in 30 provinces, including government agencies, enterprises, public institutions, colleges and universities, People’s Armed Police, communities, tourist attractions, densely populated areas, emergency services and so on, carried out thousands of training and trained tens of thousands of people. In order to improve awareness of the public’s and created high-level lecturers, the Society (CSCVA) organized nearly 5000 people’s CPR training in Lingshui, Hainan Province, and successfully challenged the Guinness Book of Records in the world, in November 2016. (See Fig. 6, Guinness Book of World Records) The CCTV News Channel reported the event on the same day.

The immediate goal of the Society is to train 1 million people within 2 years, covering activities above the county level. At the same time, the Society (CSCVA) work on the establishment of a long-term promise to ensure the quality of CPR. CPR will benefit more people if we effectively popularize this technique. CPR could save more lives, reduce death and disability, improve public health level, reduce medical and health expenses, strengthen construction of medical codes, push reformation of medical and health system and promote the social responsibility of each citizen and social progress.

## Discussion

Cardiopulmonary Resuscitation began in China as early as 1800 years ago, following a circuitous course of brilliant experimentation, serendipitous observation, slow adoption, and forgotten-then-rediscovered wisdom. It was accepted widely by neighboring countries and provides some new ideas of modern medicine. Early Chinese records described pressing the chest but weren’t clear on the specific position, depth, frequency and other details. This was clearly a precursor of CPR. Not only in China, there also have records in other ancient countries. The remarkable red ochre drawing of a mammoth in the Pindal cave in Spain, presumably of the Paleolithic period, showing a leaf-shaped dark area where the heart should be, may depict the first attempt by humans to relate disease and death to the heart. At that time a supernatural orientation to health and disease was fundamental. Intruded spirits had to be driven out by magical-religious formulas. Some authors have postulated that resuscitative efforts consisted of yelling, crying, making loud noises, and even beating the patient in order to “wake-up” the victim. This speculation regarding life in prehistoric times, however, has been based on the analysis of those interpreting evidence such as Paleolithic art. It was only with the advent of written records that any true interpretation of early medical practice was provided [15].However, in the Jin Dynasty (265 ~ 420 AD) Chinese physicians reported mouth to mouth resuscitation. In addition, to overcome hesitation toward mouth-to-mouth contact, it was suggested to ventilate through reed or bamboo tubes. Later, Andreas Vesalius published “De Humani Corporis Fabrica” which describes blowing into a tube to resuscitate an animal around the year 1000. This was a prototype of an oropharyngeal airway. Further exploration of the lengthy history of CPR and its antecedents in China can provide us new thinking and spaces to explore insights into cardiopulmonary resuscitation. And Chinese Society of Cardiothoracic and Vascular Anesthesiology will keep on implementing activities to make CPR to be effectively popularized.

## Conclusion

In conclusion, CPR in ancient China stared really early, but it developed slowly in later years. According to the history, we realized that it is necessary for us to inherit the wise of ancient people. In present circumstance, we need to set up professional CPR training organization and train full-time trainers to popularize the skill of CPR. This is also need the support of government to achieve the goal of “Healthy China 2030”.

## Data Availability

Not applicable.

## Abbreviation

CPR: Cardiopulmonary Resuscitation
CSCVA: Chinese Society of Cardiothoracic and Vascular Anesthesiology
